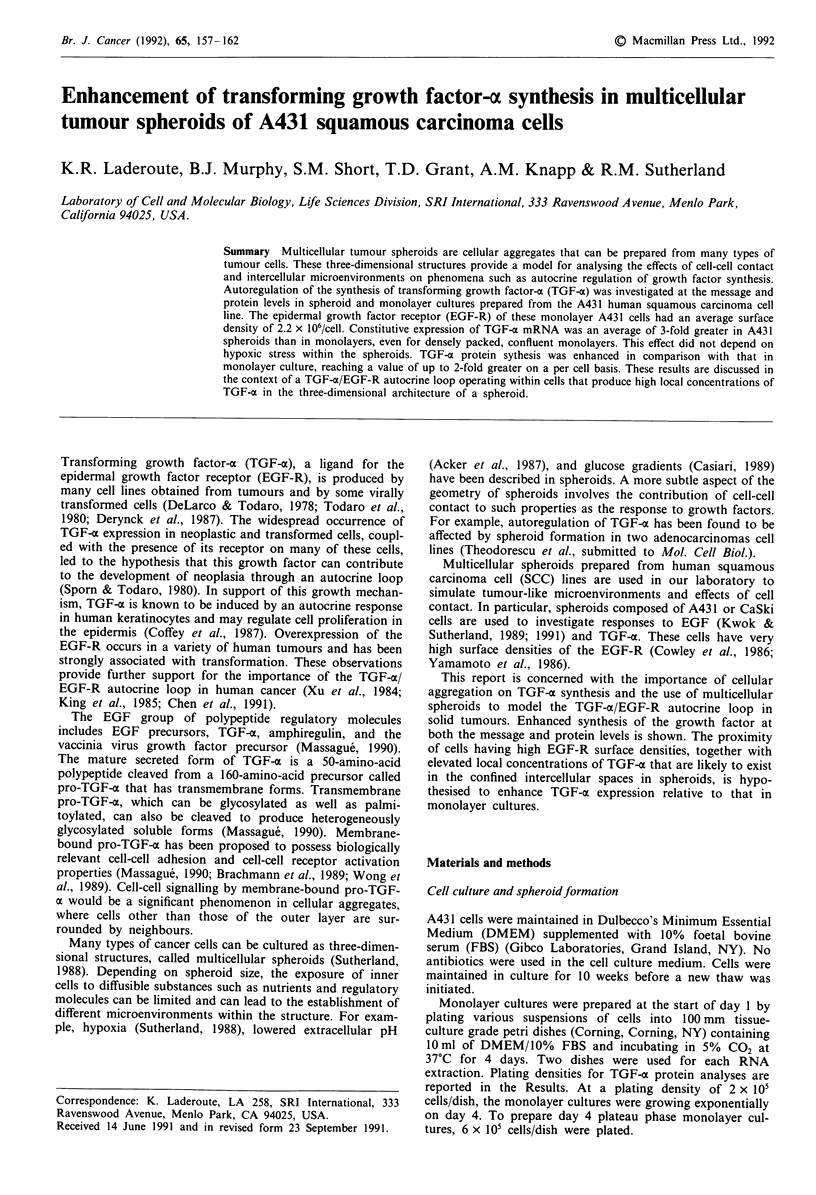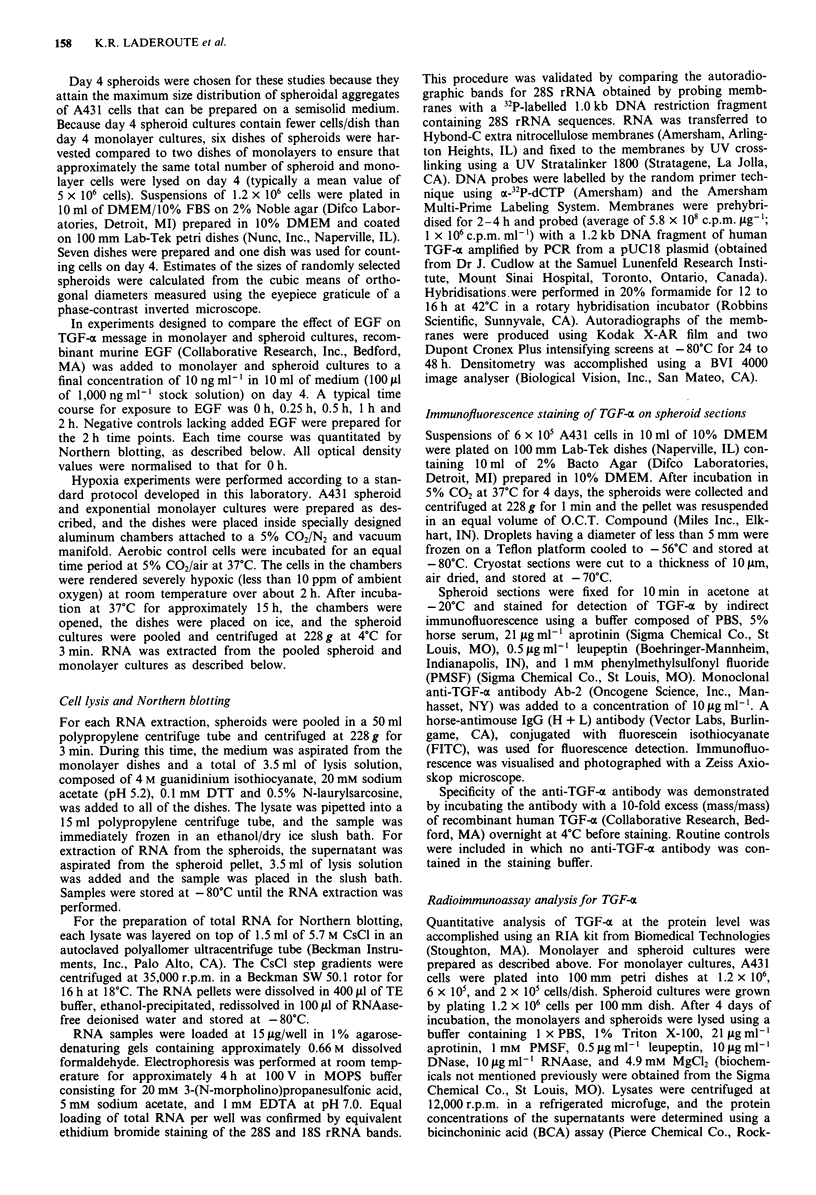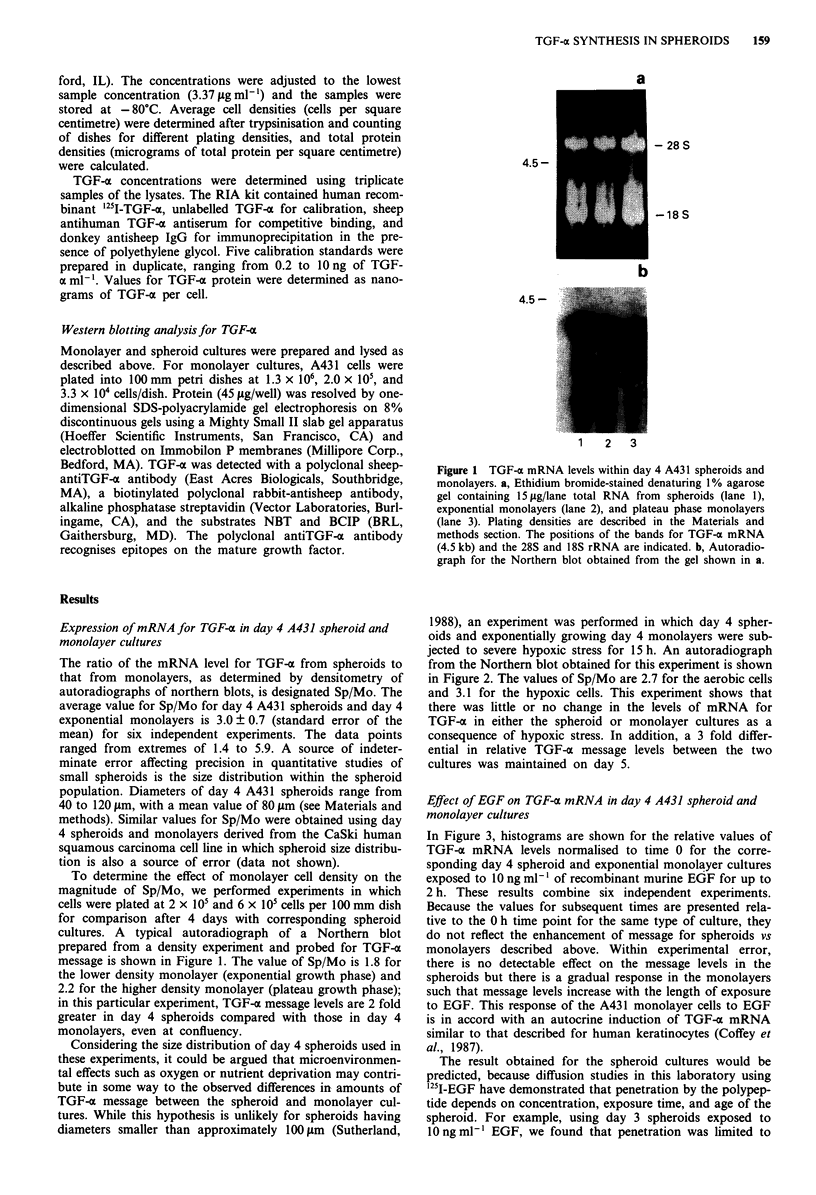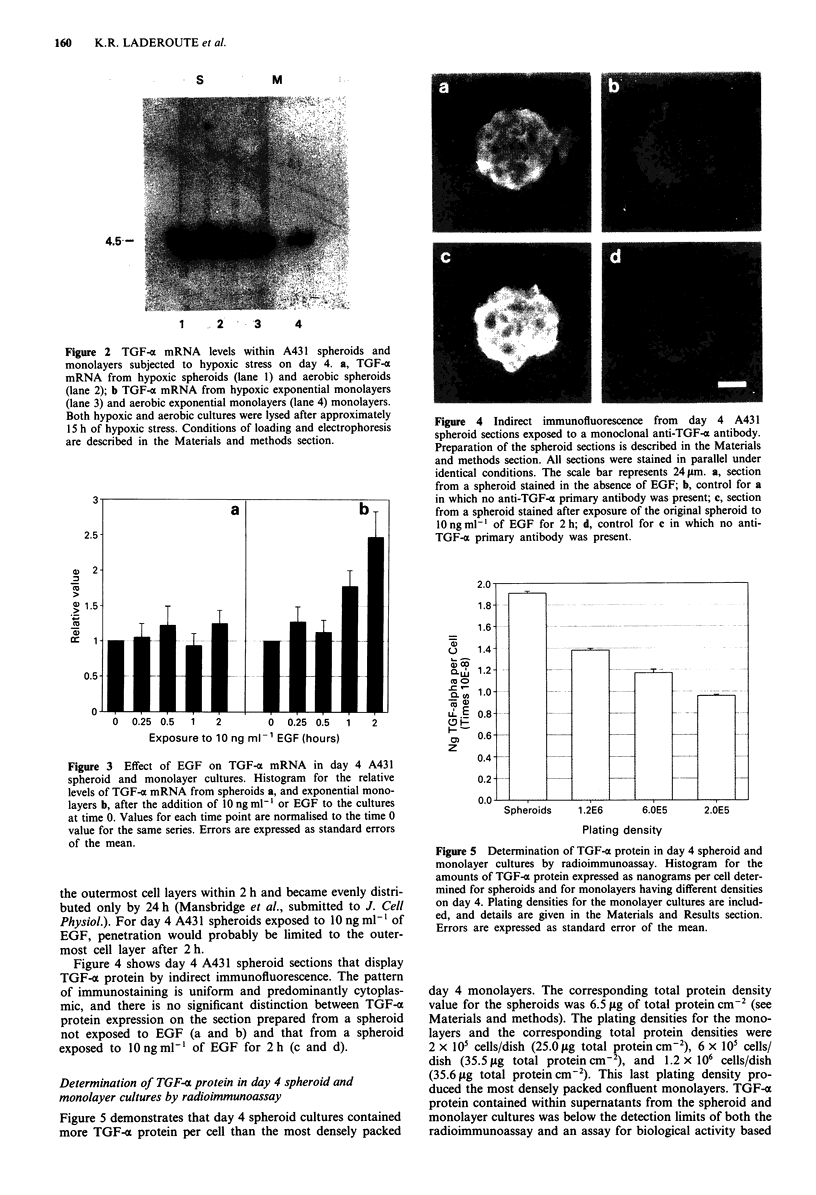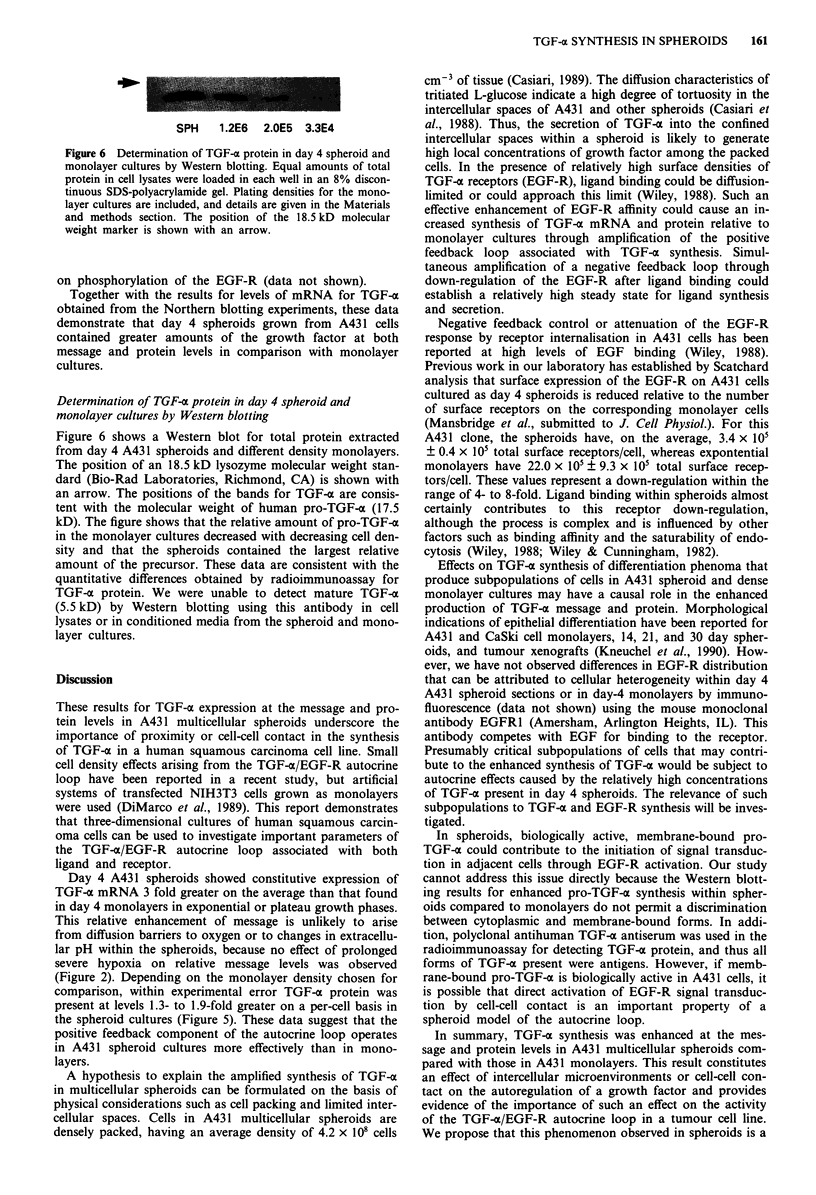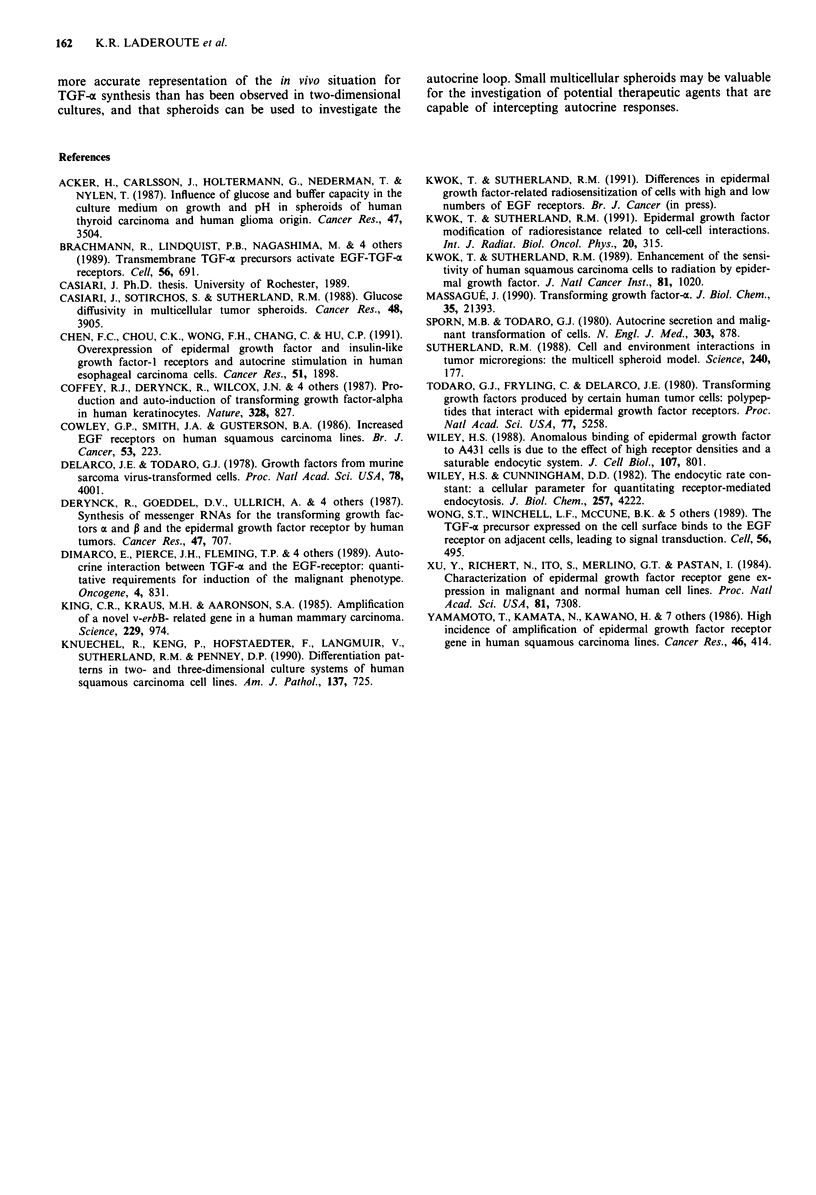# Enhancement of transforming growth factor-alpha synthesis in multicellular tumour spheroids of A431 squamous carcinoma cells.

**DOI:** 10.1038/bjc.1992.34

**Published:** 1992-02

**Authors:** K. R. Laderoute, B. J. Murphy, S. M. Short, T. D. Grant, A. M. Knapp, R. M. Sutherland

**Affiliations:** Laboratory of Cell and Molecular Biology, SRI International, Menlo Park, California 94025.

## Abstract

**Images:**


Br. J. Cancer (1992), 65, 157-162 © Macmillan Press Ltd., 1992

### Enhancement of transforming growth factor-α synthesis in multicellular tumour spheroids of A431 squamous carcinoma cells

K.R. Laderoute, B.J. Murphy, S.M. Short, T.D. Grant, A.M. Knapp & R.M. Sutherland

*Laboratory of Cell and Molecular Biology, Life Sciences Division, SRI International, 333 Ravenswood Avenue, Menlo Park, California 94025, USA.*

**Summary** Multicellular tumour spheroids are cellular aggregates that can be prepared from many types of tumour cells. These three-dimensional structures provide a model for analysing the effects of cell-cell contact and intercellular microenvironments on phenomena such as autocrine regulation of growth factor synthesis. Autoregulation of the synthesis of transforming growth factor-α (TGF-α) was investigated at the message and protein levels in spheroid and monolayer cultures prepared from the A431 human squamous carcinoma cell line. The epidermal growth factor receptor (EGF-R) of these monolayer A431 cells had an average surface density of $2.2 \times 10^6$/cell. Constitutive expression of TGF-α mRNA was an average of 3-fold greater in A431 spheroids than in monolayers, even for densely packed, confluent monolayers. This effect did not depend on hypoxic stress within the spheroids. TGF-α protein sythesis was enhanced in comparison with that in monolayer culture, reaching a value of up to 2-fold greater on a per cell basis. These results are discussed in the context of a TGF-α/EGF-R autocrine loop operating within cells that produce high local concentrations of TGF-α in the three-dimensional architecture of a spheroid.

Transforming growth factor-α (TGF-α), a ligand for the epidermal growth factor receptor (EGF-R), is produced by many cell lines obtained from tumours and by some virally transformed cells (DeLarco & Todaro, 1978; Todaro *et al.*, 1980; Derynck *et al.*, 1987). The widespread occurrence of TGF-α expression in neoplastic and transformed cells, coupled with the presence of its receptor on many of these cells, led to the hypothesis that this growth factor can contribute to the development of neoplasia through an autocrine loop (Sporn & Todaro, 1980). In support of this growth mechanism, TGF-α is known to be induced by an autocrine response in human keratinocytes and may regulate cell proliferation in the epidermis (Coffey *et al.*, 1987). Overexpression of the EGF-R occurs in a variety of human tumours and has been strongly associated with transformation. These observations provide further support for the importance of the TGF-α/EGF-R autocrine loop in human cancer (Xu *et al.*, 1984; King *et al.*, 1985; Chen *et al.*, 1991).

The EGF group of polypeptide regulatory molecules includes EGF precursors, TGF-α, amphiregulin, and the vaccinia virus growth factor precursor (Massagué, 1990). The mature secreted form of TGF-α is a 50-amino-acid polypeptide cleaved from a 160-amino-acid precursor called pro-TGF-α that has transmembrane forms. Transmembrane pro-TGF-α, which can be glycosylated as well as palmitoylated, can also be cleaved to produce heterogeneously glycosylated soluble forms (Massagué, 1990). Membrane-bound pro-TGF-α has been proposed to possess biologically relevant cell-cell adhesion and cell-cell receptor activation properties (Massagué, 1990; Brachmann *et al.*, 1989; Wong *et al.*, 1989). Cell-cell signalling by membrane-bound pro-TGF-α would be a significant phenomenon in cellular aggregates, where cells other than those of the outer layer are surrounded by neighbours.

Many types of cancer cells can be cultured as three-dimensional structures, called multicellular spheroids (Sutherland, 1988). Depending on spheroid size, the exposure of inner cells to diffusible substances such as nutrients and regulatory molecules can be limited and can lead to the establishment of different microenvironments within the structure. For example, hypoxia (Sutherland, 1988), lowered extracellular pH (Acker *et al.*, 1987), and glucose gradients (Casiari, 1989) have been described in spheroids. A more subtle aspect of the geometry of spheroids involves the contribution of cell-cell contact to such properties as the response to growth factors. For example, autoregulation of TGF-α has been found to be affected by spheroid formation in two adenocarcinomas cell lines (Theodorescu *et al.*, submitted to *Mol. Cell Biol.*).

Multicellular spheroids prepared from human squamous carcinoma cell (SCC) lines are used in our laboratory to simulate tumour-like microenvironments and effects of cell contact. In particular, spheroids composed of A431 or CaSki cells are used to investigate responses to EGF (Kwok & Sutherland, 1989; 1991) and TGF-α. These cells have very high surface densities of the EGF-R (Cowley *et al.*, 1986; Yamamoto *et al.*, 1986).

This report is concerned with the importance of cellular aggregation on TGF-α synthesis and the use of multicellular spheroids to model the TGF-α/EGF-R autocrine loop in solid tumours. Enhanced synthesis of the growth factor at both the message and protein levels is shown. The proximity of cells having high EGF-R surface densities, together with elevated local concentrations of TGF-α that are likely to exist in the confined intercellular spaces in spheroids, is hypothesised to enhance TGF-α expression relative to that in monolayer cultures.

### Materials and methods

### *Cell culture and spheroid formation*

A431 cells were maintained in Dulbecco's Minimum Essential Medium (DMEM) supplemented with 10% foetal bovine serum (FBS) (Gibco Laboratories, Grand Island, NY). No antibiotics were used in the cell culture medium. Cells were maintained in culture for 10 weeks before a new thaw was initiated.

Monolayer cultures were prepared at the start of day 1 by plating various suspensions of cells into 100 mm tissue-culture grade petri dishes (Corning, Corning, NY) containing 10 ml of DMEM/10% FBS and incubating in 5% $CO_2$ at 37°C for 4 days. Two dishes were used for each RNA extraction. Plating densities for TGF-α protein analyses are reported in the Results. At a plating density of $2 \times 10^5$ cells/dish, the monolayer cultures were growing exponentially on day 4. To prepare day 4 plateau phase monolayer cultures, $6 \times 10^5$ cells/dish were plated.

Correspondence: K. Laderoute, LA 258, SRI International, 333 Ravenswood Avenue, Menlo Park, CA 94025, USA.
Received 14 June 1991 and in revised form 23 September 1991.

Day 4 spheroids were chosen for these studies because they attain the maximum size distribution of spheroidal aggregates of A431 cells that can be prepared on a semisolid medium. Because day 4 spheroid cultures contain fewer cells/dish than day 4 monolayer cultures, six dishes of spheroids were harvested compared to two dishes of monolayers to ensure that approximately the same total number of spheroid and monolayer cells were lysed on day 4 (typically a mean value of $5 \times 10^6$ cells). Suspensions of $1.2 \times 10^6$ cells were plated in 10 ml of DMEM/10% FBS on 2% Noble agar (Difco Laboratories, Detroit, MI) prepared in 10% DMEM and coated on 100 mm Lab-Tek petri dishes (Nunc, Inc., Naperville, IL). Seven dishes were prepared and one dish was used for counting cells on day 4. Estimates of the sizes of randomly selected spheroids were calculated from the cubic means of orthogonal diameters measured using the eyepiece graticule of a phase-contrast inverted microscope.

In experiments designed to compare the effect of EGF on TGF-α message in monolayer and spheroid cultures, recombinant murine EGF (Collaborative Research, Inc., Bedford, MA) was added to monolayer and spheroid cultures to a final concentration of 10 ng $ml^{-1}$ in 10 ml of medium (100 μl of 1,000 ng $ml^{-1}$ stock solution) on day 4. A typical time course for exposure to EGF was 0 h, 0.25 h, 0.5 h, 1 h and 2 h. Negative controls lacking added EGF were prepared for the 2 h time points. Each time course was quantitated by Northern blotting, as described below. All optical density values were normalised to that for 0 h.

Hypoxia experiments were performed according to a standard protocol developed in this laboratory. A431 spheroid and exponential monolayer cultures were prepared as described, and the dishes were placed inside specially designed aluminum chambers attached to a 5% $CO_2/N_2$ and vacuum manifold. Aerobic control cells were incubated for an equal time period at 5% $CO_2$/air at 37°C. The cells in the chambers were rendered severely hypoxic (less than 10 ppm of ambient oxygen) at room temperature over about 2 h. After incubation at 37°C for approximately 15 h, the chambers were opened, the dishes were placed on ice, and the spheroid cultures were pooled and centrifuged at 228 *g* at 4°C for 3 min. RNA was extracted from the pooled spheroid and monolayer cultures as described below.

### *Cell lysis and Northern blotting*

For each RNA extraction, spheroids were pooled in a 50 ml polypropylene centrifuge tube and centrifuged at 228 *g* for 3 min. During this time, the medium was aspirated from the monolayer dishes and a total of 3.5 ml of lysis solution, composed of 4 M guanidinium isothiocyanate, 20 mM sodium acetate (pH 5.2), 0.1 mM DTT and 0.5% N-laurylsarcosine, was added to all of the dishes. The lysate was pipetted into a 15 ml polypropylene centrifuge tube, and the sample was immediately frozen in an ethanol/dry ice slush bath. For extraction of RNA from the spheroids, the supernatant was aspirated from the spheroid pellet, 3.5 ml of lysis solution was added and the sample was placed in the slush bath. Samples were stored at − 80°C until the RNA extraction was performed.

For the preparation of total RNA for Northern blotting, each lysate was layered on top of 1.5 ml of 5.7 M CsCl in an autoclaved polyallomer ultracentrifuge tube (Beckman Instruments, Inc., Palo Alto, CA). The CsCl step gradients were centrifuged at 35,000 r.p.m. in a Beckman SW 50.1 rotor for 16 h at 18°C. The RNA pellets were dissolved in 400 μl of TE buffer, ethanol-precipitated, redissolved in 100 μl of RNAase-free deionised water and stored at − 80°C.

RNA samples were loaded at 15 μg/well in 1% agarose-denaturing gels containing approximately 0.66 M dissolved formaldehyde. Electrophoresis was performed at room temperature for approximately 4 h at 100 V in MOPS buffer consisting for 20 mM 3-(N-morpholino)propanesulfonic acid, 5 mM sodium acetate, and 1 mM EDTA at pH 7.0. Equal loading of total RNA per well was confirmed by equivalent ethidium bromide staining of the 28S and 18S rRNA bands. This procedure was validated by comparing the autoradiographic bands for 28S rRNA obtained by probing membranes with a $^{32}P$-labelled 1.0 kb DNA restriction fragment containing 28S rRNA sequences. RNA was transferred to Hybond-C extra nitrocellulose membranes (Amersham, Arlington Heights, IL) and fixed to the membranes by UV cross-linking using a UV Stratalinker 1800 (Stratagene, La Jolla, CA). DNA probes were labelled by the random primer technique using α-$^{32}P$-dCTP (Amersham) and the Amersham Multi-Prime Labeling System. Membranes were prehybridised for 2–4 h and probed (average of $5.8 \times 10^8$ c.p.m. $\mu g^{-1}$; $1 \times 10^6$ c.p.m. $ml^{-1}$) with a 1.2 kb DNA fragment of human TGF-α amplified by PCR from a pUC18 plasmid (obtained from Dr J. Cudlow at the Samuel Lunenfeld Research Institute, Mount Sinai Hospital, Toronto, Ontario, Canada). Hybridisations were performed in 20% formamide for 12 to 16 h at 42°C in a rotary hybridisation incubator (Robbins Scientific, Sunnyvale, CA). Autoradiographs of the membranes were produced using Kodak X-AR film and two Dupont Cronex Plus intensifying screens at − 80°C for 24 to 48 h. Densitometry was accomplished using a BVI 4000 image analyser (Biological Vision, Inc., San Mateo, CA).

### *Immunofluorescence staining of TGF-α on spheroid sections*

Suspensions of $6 \times 10^5$ A431 cells in 10 ml of 10% DMEM were plated on 100 mm Lab-Tek dishes (Naperville, IL) containing 10 ml of 2% Bacto Agar (Difco Laboratories, Detroit, MI) prepared in 10% DMEM. After incubation in 5% $CO_2$ at 37°C for 4 days, the spheroids were collected and centrifuged at 228 *g* for 1 min and the pellet was resuspended in an equal volume of O.C.T. Compound (Miles Inc., Elkhart, IN). Droplets having a diameter of less than 5 mm were frozen on a Teflon platform cooled to − 56°C and stored at − 80°C. Cryostat sections were cut to a thickness of 10 μm, air dried, and stored at − 70°C.

Spheroid sections were fixed for 10 min in acetone at − 20°C and stained for detection of TGF-α by indirect immunofluorescence using a buffer composed of PBS, 5% horse serum, 21 μg $ml^{-1}$ aprotinin (Sigma Chemical Co., St Louis, MO), 0.5 μg $ml^{-1}$ leupeptin (Boehringer-Mannheim, Indianapolis, IN), and 1 mM phenylmethylsulfonyl fluoride (PMSF) (Sigma Chemical Co., St Louis, MO). Monoclonal anti-TGF-α antibody Ab-2 (Oncogene Science, Inc., Manhasset, NY) was added to a concentration of 10 μg $ml^{-1}$. A horse-antimouse IgG (H + L) antibody (Vector Labs, Burlingame, CA), conjugated with fluorescein isothiocyanate (FITC), was used for fluorescence detection. Immunofluorescence was visualised and photographed with a Zeiss Axioskop microscope.

Specificity of the anti-TGF-α antibody was demonstrated by incubating the antibody with a 10-fold excess (mass/mass) of recombinant human TGF-α (Collaborative Research, Bedford, MA) overnight at 4°C before staining. Routine controls were included in which no anti-TGF-α antibody was contained in the staining buffer.

### *Radioimmunoassay analysis for TGF-α*

Quantitative analysis of TGF-α at the protein level was accomplished using an RIA kit from Biomedical Technologies (Stoughton, MA). Monolayer and spheroid cultures were prepared as described above. For monolayer cultures, A431 cells were plated into 100 mm petri dishes at $1.2 \times 10^6$, $6 \times 10^5$, and $2 \times 10^5$ cells/dish. Spheroid cultures were grown by plating $1.2 \times 10^6$ cells per 100 mm dish. After 4 days of incubation, the monolayers and spheroids were lysed using a buffer containing 1 × PBS, 1% Triton X-100, 21 μg $ml^{-1}$ aprotinin, 1 mM PMSF, 0.5 μg $ml^{-1}$ leupeptin, 10 μg $ml^{-1}$ DNase, 10 μg $ml^{-1}$ RNAase, and 4.9 mM $MgCl_2$ (biochemicals not mentioned previously were obtained from the Sigma Chemical Co., St Louis, MO). Lysates were centrifuged at 12,000 r.p.m. in a refrigerated microfuge, and the protein concentrations of the supernatants were determined using a bicinchoninic acid (BCA) assay (Pierce Chemical Co., Rockford, IL). The concentrations were adjusted to the lowest sample concentration (3.37 μg ml$^{-1}$) and the samples were stored at −80°C. Average cell densities (cells per square centimetre) were determined after trypsinisation and counting of dishes for different plating densities, and total protein densities (micrograms of total protein per square centimetre) were calculated.

TGF-α concentrations were determined using triplicate samples of the lysates. The RIA kit contained human recombinant $^{125}$I-TGF-α, unlabelled TGF-α for calibration, sheep antihuman TGF-α antiserum for competitive binding, and donkey antisheep IgG for immunoprecipitation in the presence of polyethylene glycol. Five calibration standards were prepared in duplicate, ranging from 0.2 to 10 ng of TGF-α ml$^{-1}$. Values for TGF-α protein were determined as nanograms of TGF-α per cell.

### *Western blotting analysis for TGF-α*

Monolayer and spheroid cultures were prepared and lysed as described above. For monolayer cultures, A431 cells were plated into 100 mm petri dishes at $1.3 \times 10^6$, $2.0 \times 10^5$, and $3.3 \times 10^4$ cells/dish. Protein (45 μg/well) was resolved by one-dimensional SDS-polyacrylamide gel electrophoresis on 8% discontinuous gels using a Mighty Small II slab gel apparatus (Hoeffer Scientific Instruments, San Francisco, CA) and electroblotted on Immobilon P membranes (Millipore Corp., Bedford, MA). TGF-α was detected with a polyclonal sheep-antiTGF-α antibody (East Acres Biologicals, Southbridge, MA), a biotinylated polyclonal rabbit-antisheep antibody, alkaline phosphatase streptavidin (Vector Laboratories, Burlingame, CA), and the substrates NBT and BCIP (BRL, Gaithersburg, MD). The polyclonal antiTGF-α antibody recognises epitopes on the mature growth factor.

**Figure 1** TGF-α mRNA levels within day 4 A431 spheroids and monolayers. **a**, Ethidium bromide-stained denaturing 1% agarose gel containing 15 μg/lane total RNA from spheroids (lane 1), exponential monolayers (lane 2), and plateau phase monolayers (lane 3). Plating densities are described in the Materials and methods section. The positions of the bands for TGF-α mRNA (4.5 kb) and the 28S and 18S rRNA are indicated. **b**, Autoradiograph for the Northern blot obtained from the gel shown in **a**.

### Results

### *Expression of mRNA for TGF-α in day 4 A431 spheroid and monolayer cultures*

The ratio of the mRNA level for TGF-α from spheroids to that from monolayers, as determined by densitometry of autoradiographs of northern blots, is designated Sp/Mo. The average value for Sp/Mo for day 4 A431 spheroids and day 4 exponential monolayers is 3.0 ± 0.7 (standard error of the mean) for six independent experiments. The data points ranged from extremes of 1.4 to 5.9. A source of indeterminate error affecting precision in quantitative studies of small spheroids is the size distribution within the spheroid population. Diameters of day 4 A431 spheroids range from 40 to 120 μm, with a mean value of 80 μm (see Materials and methods). Similar values for Sp/Mo were obtained using day 4 spheroids and monolayers derived from the CaSki human squamous carcinoma cell line in which spheroid size distribution is also a source of error (data not shown).

To determine the effect of monolayer cell density on the magnitude of Sp/Mo, we performed experiments in which cells were plated at $2 \times 10^5$ and $6 \times 10^5$ cells per 100 mm dish for comparison after 4 days with corresponding spheroid cultures. A typical autoradiograph of a Northern blot prepared from a density experiment and probed for TGF-α message is shown in Figure 1. The value of Sp/Mo is 1.8 for the lower density monolayer (exponential growth phase) and 2.2 for the higher density monolayer (plateau growth phase); in this particular experiment, TGF-α message levels are 2 fold greater in day 4 spheroids compared with those in day 4 monolayers, even at confluency.

Considering the size distribution of day 4 spheroids used in these experiments, it could be argued that microenvironmental effects such as oxygen or nutrient deprivation may contribute in some way to the observed differences in amounts of TGF-α message between the spheroid and monolayer cultures. While this hypothesis is unlikely for spheroids having diameters smaller than approximately 100 μm (Sutherland, 1988), an experiment was performed in which day 4 spheroids and exponentially growing day 4 monolayers were subjected to severe hypoxic stress for 15 h. An autoradiograph from the Northern blot obtained for this experiment is shown in Figure 2. The values of Sp/Mo are 2.7 for the aerobic cells and 3.1 for the hypoxic cells. This experiment shows that there was little or no change in the levels of mRNA for TGF-α in either the spheroid or monolayer cultures as a consequence of hypoxic stress. In addition, a 3 fold differential in relative TGF-α message levels between the two cultures was maintained on day 5.

### *Effect of EGF on TGF-α mRNA in day 4 A431 spheroid and monolayer cultures*

In Figure 3, histograms are shown for the relative values of TGF-α mRNA levels normalised to time 0 for the corresponding day 4 spheroid and exponential monolayer cultures exposed to 10 ng ml$^{-1}$ of recombinant murine EGF for up to 2 h. These results combine six independent experiments. Because the values for subsequent times are presented relative to the 0 h time point for the same type of culture, they do not reflect the enhancement of message for spheroids *vs* monolayers described above. Within experimental error, there is no detectable effect on the message levels in the spheroids but there is a gradual response in the monolayers such that message levels increase with the length of exposure to EGF. This response of the A431 monolayer cells to EGF is in accord with an autocrine induction of TGF-α mRNA similar to that described for human keratinocytes (Coffey *et al.*, 1987).

The result obtained for the spheroid cultures would be predicted, because diffusion studies in this laboratory using $^{125}$I-EGF have demonstrated that penetration by the polypeptide depends on concentration, exposure time, and age of the spheroid. For example, using day 3 spheroids exposed to 10 ng ml$^{-1}$ EGF, we found that penetration was limited to

**Figure 2** TGF-α mRNA levels within A431 spheroids and monolayers subjected to hypoxic stress on day 4. **a**, TGF-α mRNA from hypoxic spheroids (lane 1) and aerobic spheroids (lane 2); **b** TGF-α mRNA from hypoxic exponential monolayers (lane 3) and aerobic exponential monolayers (lane 4) monolayers. Both hypoxic and aerobic cultures were lysed after approximately 15 h of hypoxic stress. Conditions of loading and electrophoresis are described in the Materials and methods section.

**Figure 3** Effect of EGF on TGF-α mRNA in day 4 A431 spheroid and monolayer cultures. Histogram for the relative levels of TGF-α mRNA from spheroids **a**, and exponential monolayers **b**, after the addition of 10 ng ml$^{-1}$ or EGF to the cultures at time 0. Values for each time point are normalised to the time 0 value for the same series. Errors are expressed as standard errors of the mean.

the outermost cell layers within 2 h and became evenly distributed only by 24 h (Mansbridge *et al.*, submitted to *J. Cell Physiol.*). For day 4 A431 spheroids exposed to 10 ng ml$^{-1}$ of EGF, penetration would probably be limited to the outermost cell layer after 2 h.

Figure 4 shows day 4 A431 spheroid sections that display TGF-α protein by indirect immunofluorescence. The pattern of immunostaining is uniform and predominantly cytoplasmic, and there is no significant distinction between TGF-α protein expression on the section prepared from a spheroid not exposed to EGF (a and b) and that from a spheroid exposed to 10 ng ml$^{-1}$ of EGF for 2 h (c and d).

*Determination of TGF-α protein in day 4 spheroid and monolayer cultures by radioimmunoassay*

Figure 5 demonstrates that day 4 spheroid cultures contained more TGF-α protein per cell than the most densely packed

**Figure 4** Indirect immunofluorescence from day 4 A431 spheroid sections exposed to a monoclonal anti-TGF-α antibody. Preparation of the spheroid sections is described in the Materials and methods section. All sections were stained in parallel under identical conditions. The scale bar represents 24 μm. **a**, section from a spheroid stained in the absence of EGF; **b**, control for **a** in which no anti-TGF-α primary antibody was present; **c**, section from a spheroid stained after exposure of the original spheroid to 10 ng ml$^{-1}$ of EGF for 2 h; **d**, control for **c** in which no anti-TGF-α primary antibody was present.

**Figure 5** Determination of TGF-α protein in day 4 spheroid and monolayer cultures by radioimmunoassay. Histogram for the amounts of TGF-α protein expressed as nanograms per cell determined for spheroids and for monolayers having different densities on day 4. Plating densities for the monolayer cultures are included, and details are given in the Materials and Results section. Errors are expressed as standard error of the mean.

day 4 monolayers. The corresponding total protein density value for the spheroids was 6.5 μg of total protein cm$^{-2}$ (see Materials and methods). The plating densities for the monolayers and the corresponding total protein densities were $2 \times 10^5$ cells/dish (25.0 μg total protein cm$^{-2}$), $6 \times 10^5$ cells/dish (35.5 μg total protein cm$^{-2}$), and $1.2 \times 10^6$ cells/dish (35.6 μg total protein cm$^{-2}$). This last plating density produced the most densely packed confluent monolayers. TGF-α protein contained within supernatants from the spheroid and monolayer cultures was below the detection limits of both the radioimmunoassay and an assay for biological activity based

**Figure 6** Determination of TGF-α protein in day 4 spheroid and monolayer cultures by Western blotting. Equal amounts of total protein in cell lysates were loaded in each well in an 8% discontinuous SDS-polyacrylamide gel. Plating densities for the monolayer cultures are included, and details are given in the Materials and methods section. The position of the 18.5 kD molecular weight marker is shown with an arrow.

on phosphorylation of the EGF-R (data not shown).

Together with the results for levels of mRNA for TGF-α obtained from the Northern blotting experiments, these data demonstrate that day 4 spheroids grown from A431 cells contained greater amounts of the growth factor at both message and protein levels in comparison with monolayer cultures.

*Determination of TGF-α protein in day 4 spheroid and monolayer cultures by Western blotting*

Figure 6 shows a Western blot for total protein extracted from day 4 A431 spheroids and different density monolayers. The position of an 18.5 kD lysozyme molecular weight standard (Bio-Rad Laboratories, Richmond, CA) is shown with an arrow. The positions of the bands for TGF-α are consistent with the molecular weight of human pro-TGF-α (17.5 kD). The figure shows that the relative amount of pro-TGF-α in the monolayer cultures decreased with decreasing cell density and that the spheroids contained the largest relative amount of the precursor. These data are consistent with the quantitative differences obtained by radioimmunoassay for TGF-α protein. We were unable to detect mature TGF-α (5.5 kD) by Western blotting using this antibody in cell lysates or in conditioned media from the spheroid and monolayer cultures.

### Discussion

These results for TGF-α expression at the message and protein levels in A431 multicellular spheroids underscore the importance of proximity or cell-cell contact in the synthesis of TGF-α in a human squamous carcinoma cell line. Small cell density effects arising from the TGF-α/EGF-R autocrine loop have been reported in a recent study, but artificial systems of transfected NIH3T3 cells grown as monolayers were used (DiMarco *et al.*, 1989). This report demonstrates that three-dimensional cultures of human squamous carcinoma cells can be used to investigate important parameters of the TGF-α/EGF-R autocrine loop associated with both ligand and receptor.

Day 4 A431 spheroids showed constitutive expression of TGF-α mRNA 3 fold greater on the average than that found in day 4 monolayers in exponential or plateau growth phases. This relative enhancement of message is unlikely to arise from diffusion barriers to oxygen or to changes in extracellular pH within the spheroids, because no effect of prolonged severe hypoxia on relative message levels was observed (Figure 2). Depending on the monolayer density chosen for comparison, within experimental error TGF-α protein was present at levels 1.3- to 1.9-fold greater on a per-cell basis in the spheroid cultures (Figure 5). These data suggest that the positive feedback component of the autocrine loop operates in A431 spheroid cultures more effectively than in monolayers.

A hypothesis to explain the amplified synthesis of TGF-α in multicellular spheroids can be formulated on the basis of physical considerations such as cell packing and limited intercellular spaces. Cells in A431 multicellular spheroids are densely packed, having an average density of $4.2 \times 10^8$ cells $cm^{-3}$ of tissue (Casiari, 1989). The diffusion characteristics of tritiated L-glucose indicate a high degree of tortuosity in the intercellular spaces of A431 and other spheroids (Casiari *et al.*, 1988). Thus, the secretion of TGF-α into the confined intercellular spaces within a spheroid is likely to generate high local concentrations of growth factor among the packed cells. In the presence of relatively high surface densities of TGF-α receptors (EGF-R), ligand binding could be diffusion-limited or could approach this limit (Wiley, 1988). Such an effective enhancement of EGF-R affinity could cause an increased synthesis of TGF-α mRNA and protein relative to monolayer cultures through amplification of the positive feedback loop associated with TGF-α synthesis. Simultaneous amplification of a negative feedback loop through down-regulation of the EGF-R after ligand binding could establish a relatively high steady state for ligand synthesis and secretion.

Negative feedback control or attenuation of the EGF-R response by receptor internalisation in A431 cells has been reported at high levels of EGF binding (Wiley, 1988). Previous work in our laboratory has established by Scatchard analysis that surface expression of the EGF-R on A431 cells cultured as day 4 spheroids is reduced relative to the number of surface receptors on the corresponding monolayer cells (Mansbridge *et al.*, submitted to *J. Cell Physiol.*). For this A431 clone, the spheroids have, on the average, $3.4 \times 10^5 \pm 0.4 \times 10^5$ total surface receptors/cell, whereas expontential monolayers have $22.0 \times 10^5 \pm 9.3 \times 10^5$ total surface receptors/cell. These values represent a down-regulation within the range of 4- to 8-fold. Ligand binding within spheroids almost certainly contributes to this receptor down-regulation, although the process is complex and is influenced by other factors such as binding affinity and the saturability of endocytosis (Wiley, 1988; Wiley & Cunningham, 1982).

Effects on TGF-α synthesis of differentiation phenoma that produce subpopulations of cells in A431 spheroid and dense monolayer cultures may have a causal role in the enhanced production of TGF-α message and protein. Morphological indications of epithelial differentiation have been reported for A431 and CaSki cell monolayers, 14, 21, and 30 day spheroids, and tumour xenografts (Kneuchel *et al.*, 1990). However, we have not observed differences in EGF-R distribution that can be attributed to cellular heterogeneity within day 4 A431 spheroid sections or in day-4 monolayers by immunofluorescence (data not shown) using the mouse monoclonal antibody EGFR1 (Amersham, Arlington Heights, IL). This antibody competes with EGF for binding to the receptor. Presumably critical subpopulations of cells that may contribute to the enhanced synthesis of TGF-α would be subject to autocrine effects caused by the relatively high concentrations of TGF-α present in day 4 spheroids. The relevance of such subpopulations to TGF-α and EGF-R synthesis will be investigated.

In spheroids, biologically active, membrane-bound pro-TGF-α could contribute to the initiation of signal transduction in adjacent cells through EGF-R activation. Our study cannot address this issue directly because the Western blotting results for enhanced pro-TGF-α synthesis within spheroids compared to monolayers do not permit a discrimination between cytoplasmic and membrane-bound forms. In addition, polyclonal antihuman TGF-α antiserum was used in the radioimmunoassay for detecting TGF-α protein, and thus all forms of TGF-α present were antigens. However, if membrane-bound pro-TGF-α is biologically active in A431 cells, it is possible that direct activation of EGF-R signal transduction by cell-cell contact is an important property of a spheroid model of the autocrine loop.

In summary, TGF-α synthesis was enhanced at the message and protein levels in A431 multicellular spheroids compared with those in A431 monolayers. This result constitutes an effect of intercellular microenvironments or cell-cell contact on the autoregulation of a growth factor and provides evidence of the importance of such an effect on the activity of the TGF-α/EGF-R autocrine loop in a tumour cell line. We propose that this phenomenon observed in spheroids is a more accurate representation of the *in vivo* situation for TGF-α synthesis than has been observed in two-dimensional cultures, and that spheroids can be used to investigate the autocrine loop. Small multicellular spheroids may be valuable for the investigation of potential therapeutic agents that are capable of intercepting autocrine responses.

### References

ACKER, H., CARLSSON, J., HOLTERMANN, G., NEDERMAN, T. & NYLEN, T. (1987). Influence of glucose and buffer capacity in the culture medium on growth and pH in spheroids of human thyroid carcinoma and human glioma origin. *Cancer Res.*, **47**, 3504.

BRACHMANN, R., LINDQUIST, P.B., NAGASHIMA, M. & 4 others (1989). Transmembrane TGF-α precursors activate EGF-TGF-α receptors. *Cell*, **56**, 691.

CASIARI, J. Ph.D. thesis. University of Rochester, 1989.

CASIARI, J., SOTIRCHOS, S. & SUTHERLAND, R.M. (1988). Glucose diffusivity in multicellular tumor spheroids. *Cancer Res.*, **48**, 3905.

CHEN, F.C., CHOU, C.K., WONG, F.H., CHANG, C. & HU, C.P. (1991). Overexpression of epidermal growth factor and insulin-like growth factor-1 receptors and autocrine stimulation in human esophageal carcinoma cells. *Cancer Res.*, **51**, 1898.

COFFEY, R.J., DERYNCK, R., WILCOX, J.N. & 4 others (1987). Production and auto-induction of transforming growth factor-alpha in human keratinocytes. *Nature*, **328**, 827.

COWLEY, G.P., SMITH, J.A. & GUSTERSON, B.A. (1986). Increased EGF receptors on human squamous carcinoma lines. *Br. J. Cancer*, **53**, 223.

DELARCO, J.E. & TODARO, G.J. (1978). Growth factors from murine sarcoma virus-transformed cells. *Proc. Natl Acad. Sci. USA*, **78**, 4001.

DERYNCK, R., GOEDDEL, D.V., ULLRICH, A. & 4 others (1987). Synthesis of messenger RNAs for the transforming growth factors α and β and the epidermal growth factor receptor by human tumors. *Cancer Res.*, **47**, 707.

DIMARCO, E., PIERCE, J.H., FLEMING, T.P. & 4 others (1989). Autocrine interaction between TGF-α and the EGF-receptor: quantitative requirements for induction of the malignant phenotype. *Oncogene*, **4**, 831.

KING, C.R., KRAUS, M.H. & AARONSON, S.A. (1985). Amplification of a novel v-*erb*B- related gene in a human mammary carcinoma. *Science*, **229**, 974.

KNUECHEL, R., KENG, P., HOFSTAEDTER, F., LANGMUIR, V., SUTHERLAND, R.M. & PENNEY, D.P. (1990). Differentiation patterns in two- and three-dimensional culture systems of human squamous carcinoma cell lines. *Am. J. Pathol.*, **137**, 725.

KWOK, T. & SUTHERLAND, R.M. (1991). Differences in epidermal growth factor-related radiosensitization of cells with high and low numbers of EGF receptors. *Br. J. Cancer* (in press).

KWOK, T. & SUTHERLAND, R.M. (1991). Epidermal growth factor modification of radioresistance related to cell-cell interactions. *Int. J. Radiat. Biol. Oncol. Phys.*, **20**, 315.

KWOK, T. & SUTHERLAND, R.M. (1989). Enhancement of the sensitivity of human squamous carcinoma cells to radiation by epidermal growth factor. *J. Natl Cancer Inst.*, **81**, 1020.

MASSAGUÉ, J. (1990). Transforming growth factor-α. *J. Biol. Chem.*, **35**, 21393.

SPORN, M.B. & TODARO, G.J. (1980). Autocrine secretion and malignant transformation of cells. *N. Engl. J. Med.*, **303**, 878.

SUTHERLAND, R.M. (1988). Cell and environment interactions in tumor microregions: the multicell spheroid model. *Science*, **240**, 177.

TODARO, G.J., FRYLING, C. & DELARCO, J.E. (1980). Transforming growth factors produced by certain human tumor cells: polypeptides that interact with epidermal growth factor receptors. *Proc. Natl Acad. Sci. USA*, **77**, 5258.

WILEY, H.S. (1988). Anomalous binding of epidermal growth factor to A431 cells is due to the effect of high receptor densities and a saturable endocytic system. *J. Cell Biol.*, **107**, 801.

WILEY, H.S. & CUNNINGHAM, D.D. (1982). The endocytic rate constant: a cellular parameter for quantitating receptor-mediated endocytosis. *J. Biol. Chem.*, **257**, 4222.

WONG, S.T., WINCHELL, L.F., MCCUNE, B.K. & 5 others (1989). The TGF-α precursor expressed on the cell surface binds to the EGF receptor on adjacent cells, leading to signal transduction. *Cell*, **56**, 495.

XU, Y., RICHERT, N., ITO, S., MERLINO, G.T. & PASTAN, I. (1984). Characterization of epidermal growth factor receptor gene expression in malignant and normal human cell lines. *Proc. Natl Acad. Sci. USA*, **81**, 7308.

YAMAMOTO, T., KAMATA, N., KAWANO, H. & 7 others (1986). High incidence of amplification of epidermal growth factor receptor gene in human squamous carcinoma lines. *Cancer Res.*, **46**, 414.